# Ensemble Machine Learning Approaches for Automated Fungal Keratitis Diagnosis Using In Vivo Confocal Microscopy Images

**DOI:** 10.1049/htl2.70051

**Published:** 2025-12-19

**Authors:** Sowmya Kamath S., Shikha Reji, Vaibhava Lakshmi, Supreetha R., Pratiksha Gawas, Veena Mayya, Manali Hazarika

**Affiliations:** ^1^ Healthcare Analytics and Language Engineering (HALE) Lab Department of Information Technology National Institute of Technology, Surathkal Mangaluru Karnataka India; ^2^ Manipal Institute of Technology, Manipal Academy of Higher Education Manipal Karnataka India; ^3^ Cornea, Cataract & Ocular Surface Services Royal Eye Infirmary ‐ University Hospitals Plymouth NHS Trust Plymouth England

**Keywords:** biomedical imaging, image processing, medical image processing

## Abstract

Fungal keratitis (FK) is a severe ocular infection that can lead to significant vision problems or blindness if not diagnosed and treated promptly. Early and accurate detection of FK is essential for effective management. Traditional diagnostic methods are often time‐consuming and require specialized laboratory resources. Recently, advances in artificial intelligence and computer vision have enabled automated diagnosis of FK using slit‐lamp images. In this article, a comprehensive evaluation of state‐of‐the‐art techniques adopted for classifying FK using in vivo confocal microscopy (IVCM) images is presented. Detailed experiments and performance evaluation of various machine learning models are systematically performed, with a focus on evaluating the effect of diverse techniques for image processing, data augmentation, hyperparameters and model finetuning to assess each model's strengths and limitations. Experiments revealed that applying green channel preprocessing with a 12‐feature set achieved 99% accuracy using Random Forest, highlighting its effectiveness in FK detection, while complex techniques like histogram modelling reduced accuracy to 64%. Robust models like AdaBoost and RUSBoost maintained high F1‐scores, demonstrating adaptability to imbalanced medical datasets, and to real‐world clinical scenarios.

## Graphical abstract

In this work, a comprehensive evaluation of state‐of‐the‐art techniques adopted for classifying fungal keratitis using in vivo confocal microscopy images is presented. Detailed experiments and performance evaluation of various machine learning models are systematically performed, with a focus on evaluating the effect of diverse techniques for image processing, data augmentation, hyperparameters and model finetuning to assess each model's strengths and limitations.

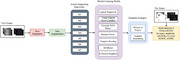

**Abbreviations**
FK: fungal keratitis;
IVCM: in vivo confocal microscopy
SVC: linear support vector classifier
kNN: K‐nearest neighbours
MoE: mixture of experts
AUROC: area under receiver operator curve
AUPRC: area under precision recall curve

## 1 Introduction

Fungal keratitis (FK) is a severe corneal infection caused by pathogens such as *Fusarium*, *Aspergillus*, and *Candida* species, typically arising when fungal spores enter the cornea through plant‐related eye injuries or due to risk factors like improper contact lens use, contaminated lens solutions, or compromised ocular immunity [1]. Individuals in agricultural occupations and contact lens users are particularly vulnerable. Clinical symptoms include persistent redness, moderate to severe pain, photophobia, tearing, and thick discharge, with a characteristic corneal ulcer appearing as a greyish‐white lesion with irregular or feathery margins that can be detected via slit‐lamp examination [2]. If untreated, the infection can rapidly progress, leading to corneal swelling, scarring, and severe vision loss, highlighting the critical need for early diagnosis and timely intervention.

The diagnosis of FK involves a comprehensive eye examination, focusing on identifying the causative fungal organism and assessing the extent of corneal involvement. Key diagnostic methods include clinical examination and corneal scraping with microbiology smear examination (MSE). Corneal scraping and MSE involve the gentle scraping of corneal tissue from the ulcerated area for examination under a microscope. Staining techniques, such as potassium hydroxide (KOH) or calcofluor white stain, are employed to visualise fungal hyphae or yeast cells in the sample, facilitating the early detection of a fungal infection [2]. Other methods of diagnosis include Culture & antimicrobial sensitivity testing and polymerase chain reaction (PCR). The fungal species causing the infection is isolated and identified by cultivating the corneal sample on a fungal‐specific medium. Since different fungal species may require distinct therapies, this step is crucial for confirming the diagnosis and selecting the most effective antifungal treatment. When traditional techniques such as MSE and culture are inconclusive, PCR testing is effective for detecting fungal DNA with improved sensitivity. Unlike MSE, PCR does not require a tissue sample from the corneal region to detect fungal structures.

Even though comprehensive diagnostics are available, these conventional FK assessment techniques present significant challenges that can often result in treatment delays with serious visual consequences. Techniques such as sensitivity testing and corneal culture can be time‐consuming, often requiring days to produce definitive results, delaying the initiation of specific antifungal treatment, increasing the risk of the infection worsening. Corneal scraping is used to collect samples which is invasive, uncomfortable for patients, and may aggravate corneal injuries [3]. Additionally, the accuracy of traditional techniques such as MSE and culture can be limited, particularly in the early stages of infection or cases with minimal fungal presence, potentially leading to misinterpretation. Advanced techniques like PCR and confocal microscopy offer more precise identification, but are expensive, require specialized equipment, and demand greater expertise, making them less accessible in resource‐limited settings. Furthermore, traditional methods often rely on expert interpretation of image samples, which can result in diagnostic discrepancies, particularly in regions with limited ophthalmological expertise. These inherent challenges in accurate diagnosis of FK and its variants have been addressed using artificial intelligence (AI) models, which are particularly effective in evaluating corneal images. However, insufficient data for training models, the proliferation of false negatives, limited generalizability of data, and variability in performance across different devices used to capture corneal images are major barriers.

A detailed review was undertaken as per PRISMA guidelines [4] to analyse existing studies on FK diagnosis using cornea imaging. Each of these works were evaluated based on performance, robustness, and adaptability across datasets. The analysis highlights strengths and limitations in preprocessing, feature extraction, and classification methods for ML and DL models. The PRISMA guidelines are followed to identify the significant previous works. Multiple databases, namely, Scopus, IEEE Xplore, ACM, Springer, and Elsevier, are reviewed exhaustively. The search terms include ([diagnosis OR detection OR prediction] AND [microbial keratitis]) OR ([FK] OR [infectious keratitis]) during the publication year range of 2020‐2024. After removing duplicates and irrelevant articles, additional filtering is carried out using predetermined inclusion/exclusion criteria. The predetermined inclusion criteria considered are publications between 2020–2024, works related infectious or fungal keratitis diagnosis/detection/prediction, works with full text availability, works relevant to AI/ML models and reporting evaluation results. Similarly, a few exclusion criteria were also considered—works containing outdated or irrelevant research, non‐english studies, covering non‐AI approaches, and those with insufficient data and duplicate studies were filtered out. The search was performed to identify all studies in which cornea imaging is used for the early diagnosis of FK which includes older and recent reviews. Only English language articles and core conferences are considered. Also, only original studies are considered, excluding the case reports and previous review/survey articles. The key contributions of this work are listed below:

- Development of a lightweight, effective machine learning (ML) framework for keratitis classification.
- Systematic evaluation of diverse preprocessing strategies across multiple ML models to assess their diagnostic performance.
- Investigation of advanced ensemble strategies, including mixture of experts and snapshot ensembling, to analyse their influence on classification accuracy and robustness.

The remainder of this article is organized as follows: Section 2 provides a comprehensive literature review of works addressing the task considered. Section 3 presents a detailed discussion on proposed methodology, including dataset details and data augmentation techniques, model design, training and optimization. In Section 4, the experiments carried out and the observations are presented, followed by conclusion and directions for future improvements.

## 2 Related Work

The advancement of AI and computer vision has significantly transformed the accuracy of medical diagnostic tasks across various domains. Numerous studies have explored the use of traditional and advanced methods for diagnosing corneal diseases, ranging from clinical examinations and laboratory techniques like MSE and culture to cutting‐edge solutions through the design and development of effective preprocessing, feature engineering, and AI models [5, 6, 7]. In this section, we provide a detailed overview of existing research on FK diagnosis, focusing on the evolution of diagnostic techniques, the application of ML and DL algorithms, and the integration of image processing methodologies. Additionally, the limitations of traditional approaches and the challenges associated with AI‐driven models, such as data scarcity, false negatives, and device variability, are discussed to highlight the need for further advancements in this domain.

A recent study [8] attempted to identify the morphological features associated with Acanthamoeba keratitis (AK) using in vivo confocal microscopy (IVCM) images. These images were graded by experienced ophthalmologists, with a focus on specific morphological characteristics of Acanthamoeba. The study is a retrospective analysis of 27 patients with clinically suspected AK who underwent IVCM imaging. Data was analysed using logistic regression, chi‐squared testing, or Fishe's exact test, with a significance threshold set at p < 0.05. The authors reported that IVCM images are superior in detecting AK compared to traditional methods, such as culture and slit‐lamp examination. This emphasizes the importance of performing IVCM before corneal scraping to maximize organism detection and suggests incorporating this into diagnostic protocols.

A study by Wu et al. [9] introduced a novel framework for automatic hyphae detection in corneal images. This framework integrates adaptive robust binary pattern (ARBP) for texture classification, support vector machine (SVM) for image classification, and line segment detection (LSD) for hyphae detection. By combining these techniques, the framework enhances the accuracy of FK diagnosis and provides a quantitative measure of infection severity through hyphal density analysis. The preprocessing techniques used by the authors do not effectively address issues such as image noise or uneven illumination. Employing more advanced contrast enhancement and noise reduction methods could have improved hyphae visibility and produced more reliable results. Tang et al. [10] proposed an ML approach to classify pathogenic fungal genera, including *Fusarium* and *Aspergillus*, from corneal confocal microscopy images. The approach uses two classification techniques, ML (decision tree (DT), PCA, LightGBM) and deep learning (DL) (fully connected network). Employing ML techniques such as SVM for texture classification and DTs, PCA, and LightGBM for fungal genus classification have also demonstrated their effectiveness in tasks such as texture analysis and feature extraction for classification.

Alquran et al. [11] proposed a ML based approach for automated corneal ulcer detection using manual and automatic feature extraction with dimensionality reduction techniques like principal component analysis (PCA) and infinite latent feature selection (ILFS). A cascaded SVM classifier was trained which achieved 93% accuracy in the task of severity grading. In the preprocessing stage, the authors employed basic histogram equalization. Utilizing more advanced techniques, such as contrast‐limited adaptive histogram equalization (CLAHE) or adaptive illumination correction, could have better preserved fine corneal details under varying lighting conditions and potentially enhanced the classification performance. Wei et al. [12] developed a ML based diagnostic model for early detection of FK using slit‐lamp images which considered key clinical features to train logistic regression, Random Forest (RF), and DT classifiers. The logistic regression model achieved the highest accuracy (90.5%) in external validation across seven ophthalmic centres. Ren et al. [13] developed a ML model using conjunctival microbiota data to classify microbial keratitis (MK). They trained a RF classifier for identifying high‐throughput 16S rDNA sequencing of distinct bacterial signatures, which achieved over 93% accuracy. Their findings suggest microbiome‐based ML models as a promising non‐invasive diagnostic tool for MK.

Malik et al. [14] developed a ML framework for eye disease classification using DT, RF, Naïve Bayes, and neural networks, achieving over 90% accuracy. It integrates structured symptom hierarchies, ICD‐10 coding, and feature selection techniques to enhance diagnostic efficiency and automate ophthalmic disease detection. Susila et al. [15] presented an automated approach for detecting corneal ulcers using image processing techniques, including data augmentation and feature extraction with grey level co‐occurrence matrix (GLCM), followed by classification using SVM, RF, and DT. The performance of these models is compared based on accuracy, F1‐score, recall, and precision, with SVM achieving the highest accuracy of 95.67%.

Wang et al. [16] presented a ML‐based prognostic model for corneal ulcer outcomes, while also leveraging DL for lesion segmentation and classification. Using multi‐centre slit lamp image datasets, the model is trained to identify five lesion types and integrates clinical data to predict ulcer perforation and visual impairment. DeepLabV3 was used for segmentation, while XGBoost and LightGBM achieved high AUC scores (up to 0.98) for prognosis prediction. They used manual ROI annotation, resizing, random cropping, and horizontal flipping as preprocessing steps but did not perform intensity normalization, contrast enhancement, and advanced augmentation. Incorporating techniques such as CLAHE, colour standardization, and denoising could enhance segmentation and classification accuracy, particularly for rare lesions like corneal descemetocele. Bustamante‐Arias et al. [17] focused on developing ML models to differentiate between healthy and pathological corneal images using spectral‐domain optical coherence tomography (SD‐OCT). They used RF model with transfer learning (TL) approaches, with TL‐SVM and TL‐RF achieving the highest classification accuracy (AUC = 0.94 and 0.92, respectively). A very small dataset was used (fewer than 100 samples), which limits the model's generalizability. Moreover, the impact of preprocessing on performance is not comprehensively evaluated. Expanding the dataset with a more diverse set of images would enable a more reliable assessment of preprocessing effectiveness and enhance the overall robustness of the model.

The detailed literature survey conducted demonstrated the effectiveness of ML techniques in early detection and classification. Various studies explored different methodologies, including texture analysis, feature extraction, and hybrid models integrating traditional classifiers like SVM, DT and RF with deep networks. Feature selection techniques such as PCA, ILFS, and GLCM improved diagnostic accuracy, with models achieving up to 95.67% accuracy. A review of existing studies reveals that only a few machine learning models have been applied to the diagnosis of FK using IVCM images. Recent research has primarily focused on deep learning approaches, however, these models are computationally intensive and require large datasets. Most studies employ minimal or no preprocessing, and none have systematically evaluated the impact of different preprocessing techniques on model performance. The effectiveness of machine learning models on limited datasets remains unexplored.

Our work addresses the limitations of existing studies by systematically evaluating various ML models for FK classification by conducting a comprehensive analysis of base ML models and ensemble approaches to identify the most effective diagnostic techniques. By optimizing image preprocessing, data augmentation strategies, and hyperparameter tuning, we enhance model generalizability and robustness to varying imaging conditions. Additionally, our study includes fine‐tuning and performance benchmarking to assess each model's strengths and limitations, enabling the selection of optimal configurations that improve diagnostic reliability while balancing computational efficiency and classification accuracy. A summary of the reviewed literature is presented in Table 1.

**TABLE 1:** Summary on ML approaches for FK detection.

Work	Disease	Data modality	Preprocessing	Methodology	Remarks
[11]	Keratitis	Fluorescein‐stained images	image augmentation, contrast enhancement, grey‐scale conversion, morphological opening, RGB to HSV colour space	PCA, SVM	Combines handcrafted and automated feature extraction. The use of a single dataset limits generalizability.
[12]	Fungal keratitis	Slit‐lamp images	Lasso regression	RF, LR, DT	Combines manual recognition with ML. Study is limited to a Chinese patient dataset.
[13]	Microbial keratitis	IVCM	No	RF	Integrates conjunctival microbiome and ML to improve microbial keratitis diagnosis. Limited by training data bias and the complexity of microbiome interactions, which affects interpretability and generalizability.
[14]	Conjunctivitis, glaucoma, and keratitis	No imaging data, Real‐time patient data from Electronic Health Records (EHR)	Data cleaning, normalization, filtering noisy data, handling missing values in EHR data	DT, RF, Naive Bayes, Neural Networks	Uses EHR data to predict various eye diseases via symptom taxonomies. Accuracy may be affected by incomplete or limited training data.
[15]	Corneal ulcer	Fluorescein staining Corneal ulcer images	Data augmentation, feature extraction by GLCM	SVM, RF, DT	Improves corneal ulcer classification with ML, but generalizability is limited by reliance on a single dataset.
[16]	Corneal ulcer	slit lamp images	Dimensionality reduction using LASSO model	XGBoost, LightGBM	Multi‐centre dataset enhances reliability and generalization, but model accuracy remains comparatively low.
[17]	Corneal pathologies	SD‐OCT	Noise reduction	RF, SVM, CNN	Simple framework; limited generalizability due to small dataset.

## 3 Materials and Methods

**FIGURE 1:**
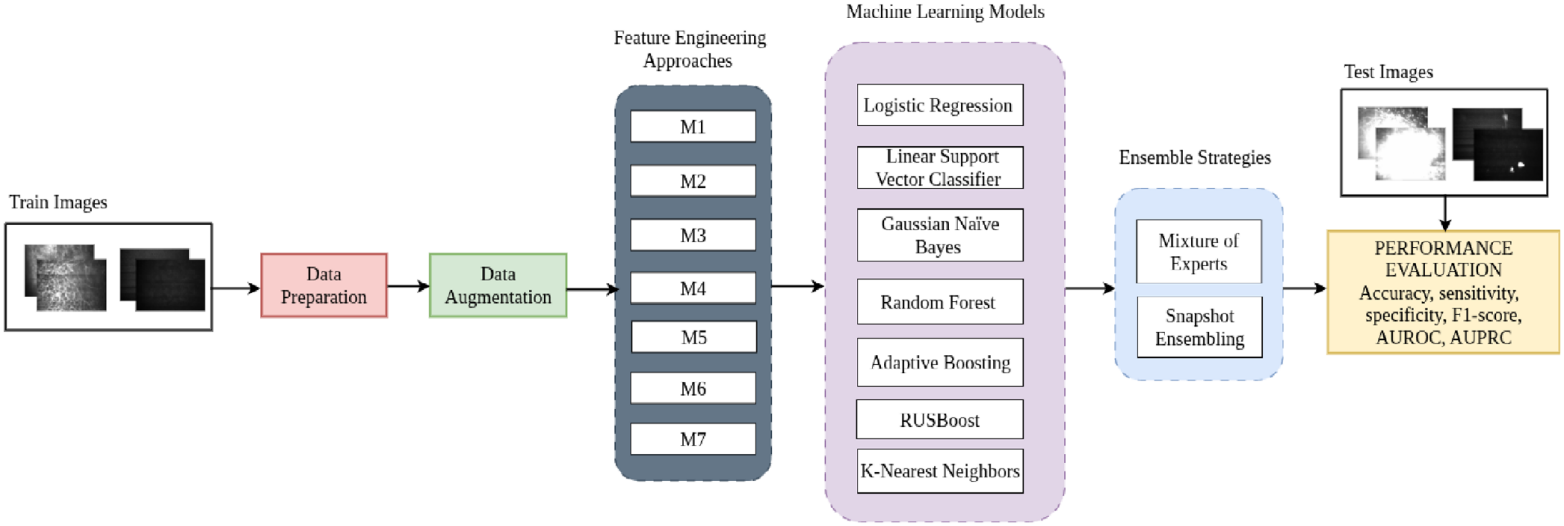
Proposed methodology (feature engineering approaches used—(i) M1: green channel, (ii) M2: green channel, image normalization, histogram modelling, (iii) M3: convolution with Gaussian mask, local maximum region extraction, (iv) M4: spatial resize, illumination equalization, denoising, adaptive contrast, colour normalization, optic disk removal, green channel, dynamics enhancing, local maxima region extraction, (v) M5: green plane, median filtering, CLAHE, vessel removal, mathematical morphology, (vi) M6: green channel, background noise removal, candidate MA extraction, non‐maximum suppression, region growing, (vii) M7: green channel, illumination and adaptive histogram equalization, greyscale normalization, and MA candidate extraction).

Figure 1 presents the workflow of the proposed framework, highlighting the preprocessing pipeline, model training, and ensemble strategies used for classification. For feature engineering, various approaches were experimented with. Each of these phases is explained in detail in the subsequent sections.

For the experimental evaluation of this study, the publicly available IVCM Keratitis Dataset [18] available for download from Figshare was utilized. The dataset contains 4001 IVCM images, classified into four categories namely normal, fungal keratitis, Acanthamoeba keratitis, and non‐specific keratitis—to support keratitis diagnosis. Given our study's focus on FK diagnosis, only fungal and non‐specific keratitis images were considered for analysis. The dataset consists of 863 images of fungal keratitis and 536 images of healthy corneas, all captured at a resolution of 768 × 576 pixels. These high‐resolution images provide detailed structural information on corneal infections, enabling robust feature extraction and model training. It serves as a valuable resource for developing and evaluating ML and DL models aimed at improving automated diagnosis. Additionally, the diversity of images allows for a comprehensive assessment of various preprocessing techniques and classification algorithms to enhance model generalization and diagnostic accuracy.

### 3.1 Data Augmentation

Several preprocessing and augmentation techniques are applied to the keratitis dataset. Preprocessing methods such as normalization, image enhancement, and resizing help standardize the input data, which is essential for training robust models. A contrast stretching method is used to enhance images obtained using Heidelberg engineering HRT‐3 confocal microscopy [9], making features critical for hyphae detection in FK more visible. To improve the accuracy of FK diagnosis, datasets containing corneal images captured under direct white light illumination [19] undergo Gaussian blur and normalization procedures. Augmentation techniques are commonly employed to enhance data variability, prevent overfitting, and artificially increase the training dataset size. The SUSTech‐SYSU dataset [20] includes high‐resolution fluorescein staining slit‐lamp images and utilizes cropping as an augmentation technique, aiding in focusing on relevant regions for corneal ulcer screening. Various datasets employ augmentation techniques such as horizontal and vertical flipping, random rotations, and random cropping. Rotations, random cropping, and contrast modulation enhance the model's ability to identify the disease from different perspectives and lighting conditions. Additionally, images are standardized to reduce noise impact using methods such as light Gaussian blur [18] and min–max Normalization [10].

The dataset, consisting of 863 fungal keratitis images and 536 healthy corneal images with a resolution of 768 × 576, underwent a comprehensive preprocessing pipeline to enhance feature representation. Various techniques were applied to refine texture and structural details while minimizing noise. Green channel extraction was employed to highlight relevant features, as it provides superior contrast in medical imaging. Median filtering reduced salt‐and‐pepper noise while preserving edges. CLAHE improved local contrast, enhancing finer details. Gaussian low‐pass filtering smoothed the image by reducing high‐frequency noise, while adaptive contrast equalization dynamically adjusted contrast across different regions. Normalization ensured consistent pixel intensity values, improving model compatibility, and illumination equalization corrected uneven lighting for better feature visibility. Additional preprocessing steps included resizing, application of Gaussian blur filters for noise reduction, histogram equalization for contrast enhancement, and background subtraction to eliminate irrelevant details. Colour space transformations, such as RGB to greyscale conversion, were also applied to ensure uniform feature extraction across different models.

**FIGURE 2:**
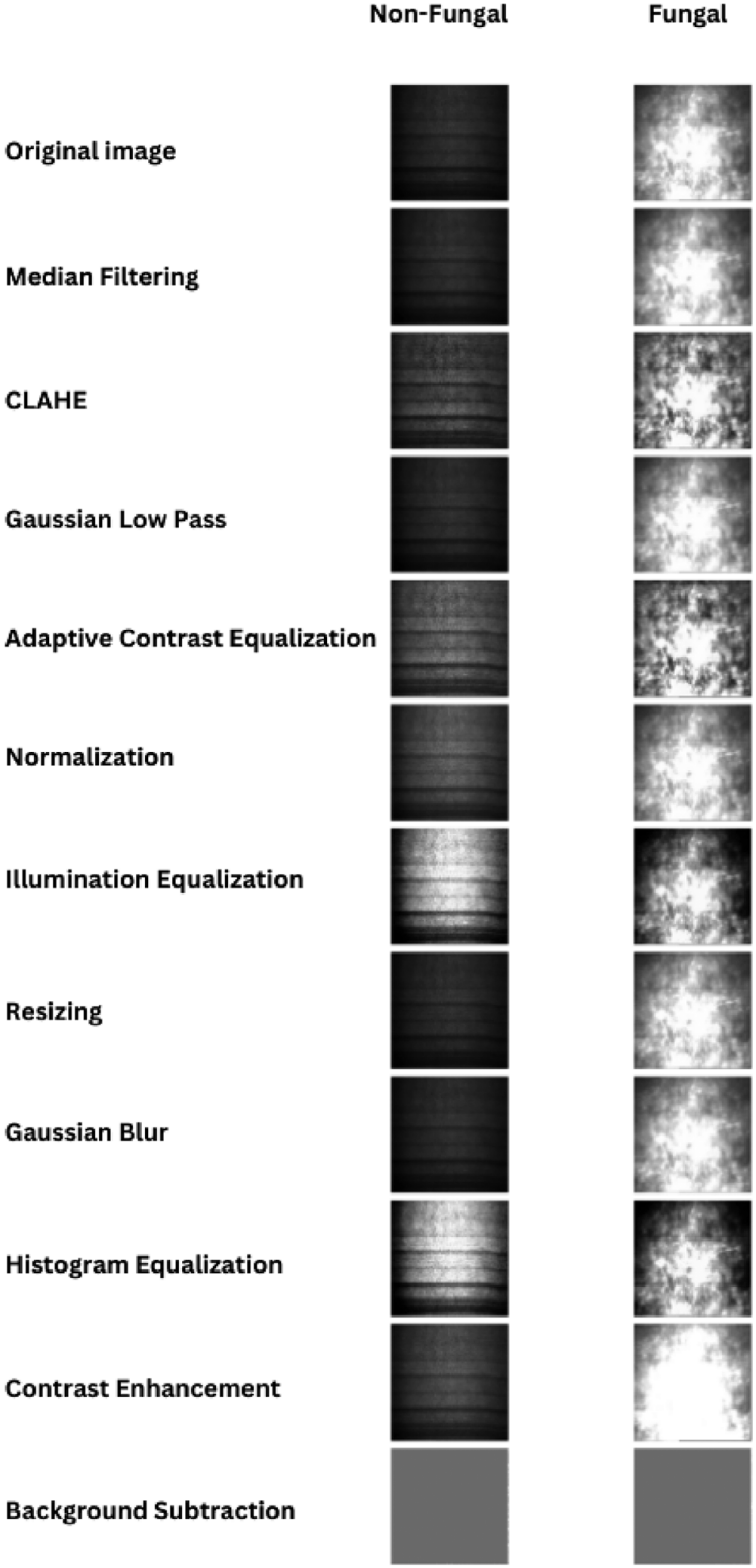
Effect of different preprocessing techniques on sample non‐fungal and fungal images from the IVCM dataset.

Figure 2 provides a detailed overview of the preprocessing techniques applied to the IVCM dataset considered for our experimental study. In the figure, the first column represents non‐fungal images, while the second column represents fungal images. The first row shows the original images before any preprocessing technique is applied, and the remaining rows display the preprocessed images after each technique was applied. Preprocessing techniques are applied to images to enhance visual quality and facilitate feature extraction. Median filtering smooths the image by removing noise while preserving edges. CLAHE [9] enhances local contrast without over‐amplifying noise, improving visibility where fungal structures are more prominent. Gaussian low‐pass filtering smooths the image by reducing high‐frequency noise. Adaptive contrast equalization [21] dynamically adjusts contrast, highlighting details in both bright and dark areas, thereby improving feature definition in fungal images. Normalization [22] scales intensity values for consistency. Illumination equalization ensures uniform lighting, aiding feature extraction. Resizing standardizes image dimensions for uniformity. Gaussian blur [19] reduces high‐frequency noise. Histogram equalization redistributes intensity values to improve contrast. Contrast enhancement highlights important structures; in fungal images, it clarifies patterns, though excessive enhancement may introduce artefacts. Background subtraction isolates the main subject by removing unnecessary background details.

### 3.2 ML Models

For the classification of IVCM images into fungal and non‐fungal categories, a range of classifiers was employed, each tailored to the specific characteristics of the extracted feature sets. Since ML models depend on diverse feature extraction techniques, capturing detailed structural and textural aspects of images was essential. The extracted features included shape attributes (e.g., area, circularity, length‐to‐width ratio, eccentricity), intensity‐based features (e.g., pixel intensity, brightness), texture features (e.g., cross‐section profiles, difference of Gaussian), and saliency‐based features (e.g., edge detector, boundary analysis). These features provided a comprehensive representation of image content, enabling accurate classification by the various ML algorithms. The models included Gaussian Naïve Bayes [23], logistic regression [24], SVM [25], k‐nearest neighbours (kNN) [26], RF [27], AdaBoost [28] and RUSBoost [29]. A summary of the model parameters used in all these seven models is presented in Table 2.

**TABLE 2:** Summary of ML model parameters.

Classifier	Parameter	Value	Selection method
**Logistic regression**	max_iter	1000	Increased for convergence
	Solver	lbfgs	sklearn default
	Penalty	l2	sklearn default
**Naive Bayes**	var_smoothing	$1\times 10^{-9}$	sklearn default
	Priors	None	sklearn default
**RF**	n_estimators	100	sklearn default
	max_depth	None	sklearn default
	min_samples_split	2	sklearn default
	Criterion	gini	sklearn default
**AdaBoost**	n_estimators	50	sklearn default
	Learning_rate	1.0	sklearn default
	Algorithm	SAMME.R	sklearn default
**RUSBoost**	n_estimators	50	Imbalanced‐learn default
	Learning_rate	1.0	Imbalanced‐learn default
	Sampling_strategy	Auto	Imbalanced‐learn default
**KNN**	n_neighbours	5	sklearn default
	Weights	Uniform	sklearn default
	Metric	Minkowski	sklearn default
**Linear SVC**	Kernel	Linear	Fixed for linearity
	Probability	True	For ROC/PR curves
	C	1.0	sklearn default

1. *Logistic regression (LR)*: LR is a linear model that estimates the probability of an input belonging to a particular class using the sigmoid function, which maps values to a range between 0 and 1. It employs the logit function to transform input features into probabilities, making it effective for binary classification tasks. To prevent overfitting, L2 regularization (ridge regression) is applied by default, penalizing large coefficient values and ensuring better generalization. Additionally, setting max_iter = 1000 allows the model to undergo sufficient iterations for convergence, improving stability and accuracy. However, LR assumes a linear relationship between features and the log‐odds of the target variable, which may not hold for complex datasets. It also struggles with highly non‐linear decision boundaries unless combined with feature engineering or kernel methods. The model is also sensitive to outliers which can significantly impact performance, and it may not be ideal for high‐dimensional data.
2. *Linear support vector classifier (SVC)*: It constructs a hyperplane that maximizes the margin between fungal and non‐fungal image categories, using a linear kernel to efficiently separate data points in high‐dimensional space. This makes it computationally efficient for large datasets. To handle class imbalance, the class weighting is adjusted, ensuring fair representation of minority classes. However, unlike other SVM variants, linear SVC does not inherently provide probability estimates. Instead, additional techniques like Platt scaling are required for probabilistic outputs. While effective for linearly separable data, some limitations exist for non‐linear patterns, making it unsuitable for complex datasets. Additionally, its performance can be sensitive to regularization parameter used, requiring careful tuning for optimal results.
3. *Gaussian Naïve Bayes (GNB)*: GNB is built on the assumption that input features are independent and follow a normal distribution, making it computationally efficient for classification tasks. It models pixel intensity distributions using a Gaussian probability function and calculates the likelihood of an input belonging to a particular class using Bayes theorem. The class with the highest posterior probability is selected as the final prediction. The model is particularly effective for datasets with moderate‐dimensional feature vectors due to its closed‐form probability estimations. However, in case of highly correlated features, the assumption of feature independence can result in performance degradation.
4. *Random Forest (RF)*: RF is an ensemble learning method that improves classification robustness by constructing multiple DTs using bootstrapped datasets and averaging their predictions. It mitigates overfitting by randomly selecting feature subsets for each tree, reducing correlation among trees and enhancing generalization. The number of estimators (trees) was set to 100, ensuring a balance between performance and computational efficiency. Additionally, the maximum tree depth was carefully adjusted to prevent overfitting while maintaining sufficient learning capability.
5. *Adaptive boosting*: AdaBoost is an ensemble learning technique that improves classification performance by iteratively reweighting misclassified instances. It combines multiple weak learners, typically decision stumps, into a strong classifier by adjusting their weights based on errors in previous iterations. In our implementation, 50 decision stumps were used as base estimators, with a learning rate of 1.0 to control the influence of each weak learner on the final prediction, ensuring a balance between adaptability and stability.
6. *RUSBoost*: RUSBoost is an extension of AdaBoost that incorporates random undersampling to handle class imbalance by reducing the majority class, thereby improving classification performance on minority class samples, such as fungal keratitis images. By maintaining a balanced dataset, the model enhances sensitivity to underrepresented cases. In our implementation, 50 base learners were used, with an optimized learning rate to ensure a stable balance between classification accuracy and model generalization.
7. *K‐nearest neighbours*: The KNN algorithm was implemented as a non‐parametric model for classifying FK images based on feature similarity with their nearest neighbours. KNN operates by assigning a class label to an input image based on the majority class of its k closest training samples, making it a simple yet effective approach for pattern recognition. Distance weighting was applied to prioritize closer neighbours, ensuring that nearer samples have a greater influence on the classification decision. Euclidean distance was chosen as the primary distance metric for feature comparison, as it effectively measures similarity in high‐dimensional feature spaces. The choice of k was optimized to balance bias‐variance trade‐off, preventing overfitting while maintaining classification accuracy.

### 3.3 ML Model Ensembling Strategies

To enhance classification performance, model ensembling strategies, including mixture of experts [30] and snapshot ensembling [31], were explored. The mixture of experts approach combines multiple specialized models, each trained to handle different aspects of the dataset, and dynamically selects the most relevant model for a given input. This strategy improves decision‐making by leveraging the strengths of diverse classifiers. Snapshot Ensembling, on the other hand, captures multiple snapshots of a single model at different training stages and averages their predictions, effectively simulating an ensemble without the need for multiple separate models. These techniques were evaluated to assess their impact on improving model robustness, reducing generalization error, and enhancing accuracy. A summary of the parameters used in the ensembling approaches is listed in Table 3.

1. *Mixture of experts (MoE)*: This framework enhances classification by combining multiple ML models by employing a gating network to dynamically assign weights to each expert's prediction. Instead of relying on a single model, MoE leverages the strengths of multiple classifiers, making it highly adaptive to different feature complexities. In this implementation, a multi‐layer perceptron (MLP) classifier acts as the gating network, learning to determine the most relevant expert for each input based on feature characteristics. This dynamic selection process allows MoE to perform robustly across varying preprocessing and feature extraction techniques. The MoE framework utilizes multiple base classifiers as experts, each contributing uniquely to the ensemble which are LR, Linear SVC, NB, RF, AdaBoost, RUSBoost and kNN. These experts together form a diverse decision‐making framework that the MoE gating network integrates dynamically. The MLP consists of one hidden layer with ten neurons, enabling it to capture non‐linear interactions between features. The activation function used is ReLU (rectified linear unit), which ensures stable gradients and prevents vanishing activations during training. The network was trained for 500 epochs, allowing it to fine‐tune the weight assignments and improve expert selection.
2. *Snapshot ensembling (SE)*: SE is an efficient ensemble learning technique that improves classification performance by leveraging multiple models trained within a single training cycle. Instead of training separate models independently, this method utilizes cyclic learning rate scheduling to force a single neural network to converge to multiple local minima, capturing diverse decision boundaries. By periodically saving model snapshots at different points in the optimization process, snapshot ensembling creates an ensemble of diverse yet computationally efficient classifiers. Here too, the MLP classifier serves as the base model, trained using cyclic learning rates to ensure dynamic exploration of different optima. The snapshots taken at scheduled intervals act as independent experts, and their outputs are averaged during inference to form a robust final prediction. This method enhances generalization by preventing overfitting to a single solution and leveraging diverse perspectives captured at different training stages. The ensemble consists of five snapshot models, each contributing a unique decision boundary. The final prediction is obtained by averaging the outputs of all snapshots, ensuring stability and improved predictive accuracy. The model architecture comprises an input layer that takes feature input from the dataset, two fully connected hidden layers with 64 and 32 neurons respectively using ReLU activation, and a single output neuron with a sigmoid activation for binary classification.

**TABLE 3:** Summary of model parameters used in ensemble approaches.

Method	Parameter	Value	Purpose
Snapshot ensembling	snapshots	5	Number of models
	epochs	10	Per snapshot
	batch_size	32	Training batch
	base_lr	0.001	Min learning rate
	max_lr	0.01	Max learning rate
	step_size	5	Cycle length
	hidden_layer_1	64	First dense layer
	hidden_layer_2	32	Second dense layer
MoE (mixture of experts)	hidden_layers	(10,)	Gating network
	max_iter	500	MLP iterations
	activation	relu	Activation function
General	test_size	0.2	Train‐test split
	random_state	42	Reproducibility

## 4 Experiments and Results

The implementation of the proposed evaluation was carried out using Python [3.12.4], with key libraries including TensorFlow [2.17.0], Keras [3.5.0] and Scikit‐learn [1.5.0] for ML and DL tasks. Data processing and visualization were performed using NumPy [1.26.4], Pandas [2.2.2], Matplotlib [3.9.2] and Seaborn [0.13.2]. Additional libraries such as OpenCV [4.10.0] and PIL were used for image processing. A virtual environment (Conda) was used to ensure dependency management and package compatibility. The experimental setup was designed to apply ML techniques on IVCM Keratitis dataset. Firstly, several processes were applied to enhance image quality to boost model performance. Initially, the dataset underwent preprocessing, including resizing, normalization, and various augmentation techniques to improve image quality and diversity. For ML‐based approaches, feature extraction was performed using green channel analysis and specific image characteristics relevant to FK detection. Extracted features were then used to train models such as RF, AdaBoost and RUSBoost. These models were evaluated based on their ability to differentiate FK from other conditions, considering key performance metrics to assess their effectiveness. The DL models were trained with the number of epochs ranging from 5 to 10, batch sizes (commonly set to 32) and learning rates ranging from 0.001 to 0.03, had been applied to the each model's architecture. Standard evaluation metrics were considered to measure the performance of different models in classifying between fungal and normal eye images. The main objective of this comprehensive evaluation is to demonstrate the robustness across various feature extraction and advanced preprocessing methods.

### 4.1 Evaluation Metrics

Several evaluation metrics were utilized to assess the performance of DL techniques for the task of FK prediction. Accuracy, sensitivity, specificity, precision, and F1‐score are essential for evaluating models in diagnostic applications. Sensitivity and specificity measure the model's ability to correctly identify positive and negative cases, respectively, while accuracy reflects the model's overall reliability. Precision assesses the reliability of positive predictions, and the F1‐score provides a balanced metric by combining precision and sensitivity, which is particularly crucial for handling imbalanced datasets. Other metrics, such as the AUROC (area under receiver operator curve) and AUPRC (area under precision recall curve) provide deeper insights into the trade‐offs between sensitivity and specificity. Additionally, training accuracy, training loss, validation accuracy, and validation loss were used to evaluate the effectiveness of DL techniques.

Accuracy is given by the ratio of correctly categorized instances to all instances in the dataset is known as accuracy. It is a general measure to assess model performance computed as per Equation (1), where, TP is true positives, TN is true negatives, FP is false positives, and FN is false negatives. Specificity (Equation 2) is calculated as the ratio of actual negative instances to the correctly identified instances, and is also known as the true negative rate. Sensitivity (Equation 3) is calculated as the ratio of actual positive instances to the actual positives that are correctly identified. F1‐score (Equation 4) provides a balance between the precision and recall as the harmonic mean, and is particularly useful in case of class imbalance.

(1) $$\text{Accuracy}=\frac{\text{TP}+\text{TN}}{\text{TP}+\text{TN}+\text{FP}+\text{FN}}$$

(2) $$\text{Specificity}=\frac{\text{TN}}{\text{TN}+\text{FP}}$$

(3) $$\text{F1-score}=2\times \frac{\text{Precision}\times \text{Recall}}{\text{Precision}+\text{Recall}}$$

(4) $$\text{Sensitivity}=\frac{\text{TP}}{\text{TP}+\text{FN}}$$

AUROC (Equation 5) measures the capacity of a binary classifier to classify between classes. It represents the area under the ROC curve, which plots the true positive rate (sensitivity) against the false positive rate (1 ‐ specificity) at different thresholds. AUPRC (Equation 6) measures the area under the precision–recall curve, which plots precision versus recall at various thresholds and is particularly useful for imbalanced datasets. Here, $\text{Precision}=\frac{\text{TP}}{\text{TP}+\text{FP}}$ and $\text{Recall}=\frac{\text{TP}}{\text{TP}+\text{FN}}$. AUROC integrates the true positive rate (TPR) as a function of the false positive rate (FPR) over the interval [0, 1] representing the total area under the ROC curve, while, AUPRC integrates precision as a function of recall over the range [0, 1], measuring the area under the precision–recall curve.

(5) $$\text{AUROC}=\int_{0}^{1}\text{TPR}\left(\text{FPR}\right)\;d\left(\text{FPR}\right)$$

(6) $$\text{AUPRC}=\int_{0}^{1}\text{Precision}\left(\text{Recall}\right)\;d\left(\text{Recall}\right)$$

The model's performance on the training dataset is calculated by training accuracy (Equation 7), which shows the percentage of accurate predictions the model made throughout the training process. Training loss gives the measure of error made by the model during the training phase which in turn demonstrates the degree to which the model's predictions and the actual label values agree. It is computed as per Equation (8), where, N represents the number of training instances, $y_{i}$ is the actual label for instance i, ${\hat{y}}_{i}$ is the predicted label for instance i, L is the loss function (e.g., cross‐entropy loss). The model's performance on the validation dataset is calculated by validation accuracy, which shows the percentage of accurate predictions the model made throughout the validation process. In order to detect possible overfitting for the model, validation loss quantifies the model's error on the validation dataset. It is computed as per Equation (10), where, M is the total number of validation instances, $y_{j}$ is the actual label for instance j, ${\hat{y}}_{j}$ is the predicted label for instance j and L is the loss function (e.g., cross‐entropy loss).

(7) $$\text{Training accuracy}=\frac{\text{Correct predictions on training set}}{\text{Total instances in training set}}$$

(8) $$\text{Training loss}=\frac{1}{N}\sum_{i=1}^{N}L\left(y_{i},{\hat{y}}_{i}\right)$$

(9) $$\text{Validation accuracy}=\frac{\text{Correct predictions on validation set}}{\text{Total instances in validation set}}$$

(10) $$\text{Validation loss}=\frac{1}{M}\sum_{j=1}^{M}L\left(y_{j},{\hat{y}}_{j}\right)$$

### 4.2 Results and Observations

Table 4 provides an overview of seven different model configurations, each incorporating distinct combinations of preprocessing and feature extraction techniques. These variations aim to assess the impact of different preprocessing strategies on model performance. The seven models are designed with distinct preprocessing and feature extraction techniques to enhance classification accuracy. M1 utilizes the green channel for feature extraction, focusing on 12 attributes such as area, degree of circularity, and length‐to‐width ratio. M2 extends preprocessing with image normalization and histogram modelling, incorporating shape, colour, brightness, and contrast‐based features. M3 applies convolution with a Gaussian mask and local maximum region extraction, emphasizing features like increasing and decreasing ramp height, top width, peak width, and peak height. M4 integrates multiple preprocessing techniques, including spatial resizing, illumination equalization, denoising, adaptive contrast adjustment, colour normalization, optic disk removal, and dynamic enhancement, with extracted features such as relative area, elongation, eccentricity, circularity, rectangularity, and solidity. M5 employs green plane extraction, median filtering, CLAHE, vessel removal, and mathematical morphology to derive features like pixel intensity, standard deviation, and six difference of Gaussian (DoG) measures. M6 incorporates background noise removal, candidate microaneurysm (MA) extraction, non‐maximum suppression, and region growing, extracting shape‐based, intensity‐based, and SBF‐based features. Lastly, M7 applies illumination and adaptive histogram equalization, greyscale normalization, and MA candidate extraction, focusing on cross‐section profile analysis, local cross‐section transformation, and local saliency analysis for feature extraction.

**TABLE 4:** Experiments conducted with various preprocessing and feature extraction techniques.

Model	Preprocessing techniques	Feature set
M1	Green channel	12 features including area, degree of circularity, and length‐to‐width ratio.
M2	Green channel, image normalization, histogram modelling.	Features based on shape, colour, brightness, and contrast.
M3	Convolution with Gaussian mask, local maximum region extraction.	Increasing and decreasing ramp height, top width, peak width, peak height, etc.
M4	Spatial resize, illumination equalization, denoising, adaptive contrast, colour normalization, optic disk removal, green channel, dynamics enhancing, local maxima region extraction.	Relative area, elongation, eccentricity, circularity, rectangularity, solidity.
M5	Green plane, median filtering, CLAHE, vessel removal, mathematical morphology.	Features including pixel's intensity, standard deviation, six difference of Gaussian.
M6	Green channel, background noise removal, candidate MA extraction, non‐maximum suppression, region growing.	Shape‐based features, intensity‐based features, SBF‐based features.
M7	Green channel, illumination and adaptive histogram equalization, greyscale normalization, MA candidate extraction.	Features based on cross‐section profile, local cross section transformation, local saliency analysis, etc.

**FIGURE 3:**
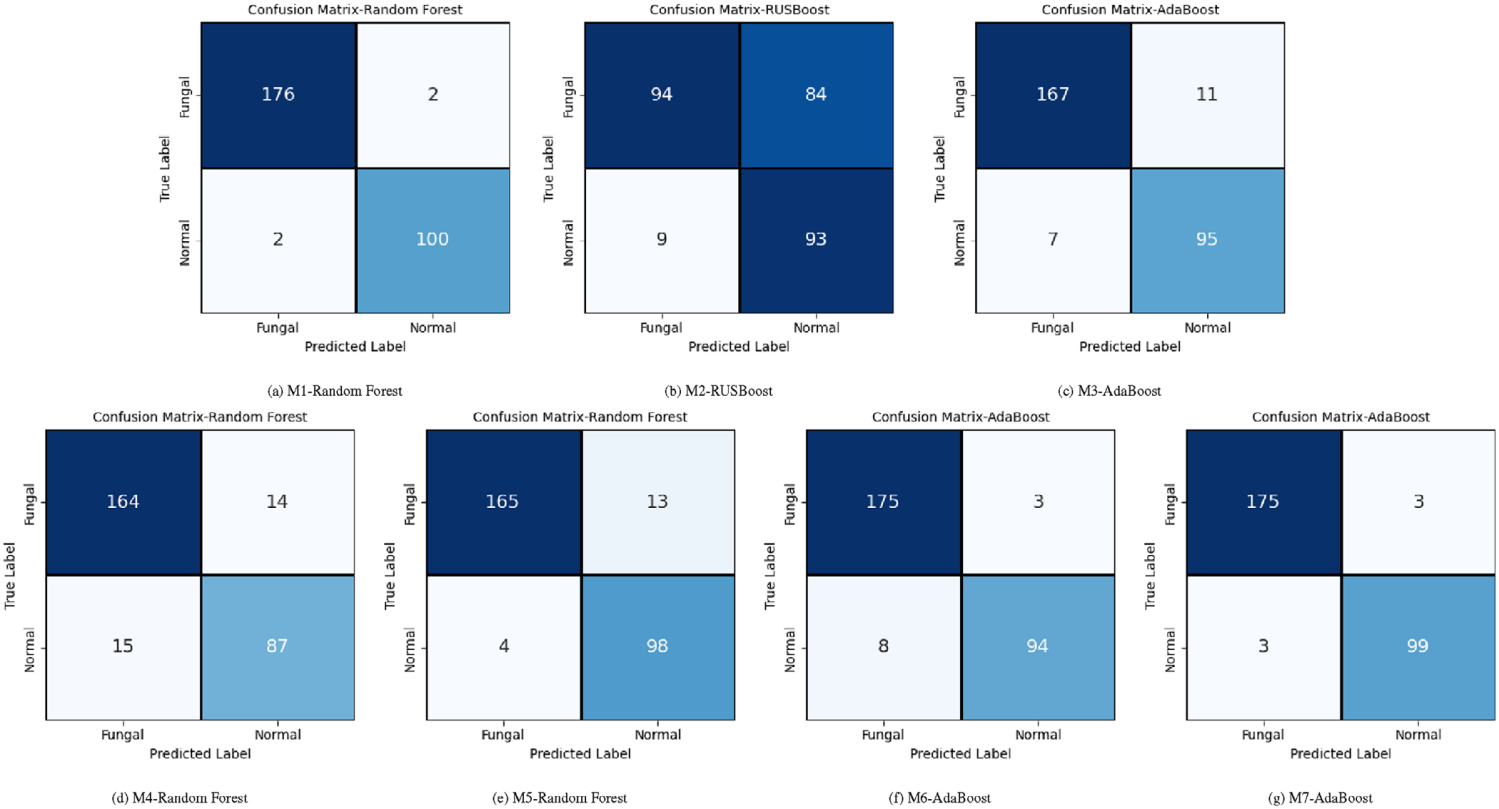
Confusion matrices of the top‐performing classifiers in model groups M1–M7 used in the experiments: (a) Random Forest for M1, (b) RUSBoost for M2, (c) AdaBoost for M3, (d) Random Forest for M4, (e) Random Forest for M5, (f) AdaBoost for M6, and (g) AdaBoost for M7.

The classification performance of Approaches 1–7 listed in Table 4, evaluated using seven ML classifiers, and that of the ensemble approaches, mixture of experts and snapshot ensembling, is presented in Table 5. Figure 3 presents confusion matrices for the top‐performing classifier in each model group (M1–M7), selected based on their AUROC values. In cases where two classifiers have the same AUROC, accuracy is used as the tiebreaker to determine the final selection.

**TABLE 5:** Performance of ML Models for the M1 to M7 combinations (details of combinations in preprocessing and feature extraction methods applied are listed in Table 4).

			F1‐score					
Model	Classifier	Accuracy	Normal	Fungal	Sensitivity	Specificity	AUROC	AUPRC
M1	Logistic regression	96%	0.94	0.97	0.92	0.98	0.97	0.97
	Linear SVC	95%	0.93	0.96	0.94	0.96	0.99	0.99
	GNB	93%	0.90	0.94	0.93	0.92	0.94	0.95
	Random Forest	**99%**	**0.98**	**0.99**	**0.98**	**0.99**	**1.0**	**1.0**
	AdaBoost	98%	**0.98**	**0.99**	**0.98**	0.98	0.99	0.98
	RUSBoost	98%	**0.98**	**0.99**	0.97	**0.99**	0.99	0.99
	KNN	92%	0.89	0.93	0.92	0.92	0.95	0.93
	Mixture of experts	98%	0.98	0.99	0.98	0.98	1.0	1.0
	Snapshot ensembling	90%	0.85	0.92	0.83	0.93	0.93	0.88
M2	Logistic regression	64%	0.00	**0.78**	0.00	**1.00**	0.43	0.34
	Linear SVC	64%	0.00	**0.78**	0.00	**1.00**	0.49	0.35
	GNB	46%	0.53	0.37	**0.83**	0.25	0.47	0.32
	Random Forest	65%	0.54	0.72	0.56	0.71	0.70	0.53
	AdaBoost	66%	0.48	0.74	0.44	0.78	0.70	0.53
	RUSBoost	66%	**0.62**	0.69	0.75	0.61	**0.73**	0.53
	KNN	**68%**	0.57	0.74	0.59	**0.73**	0.71	**0.59**
	Mixture of experts	64%	0.52	0.71	0.54	0.69	0.69	0.51
	Snapshot ensembling	64%	0.0	0.78	0.0	1.00	0.50	0.68
M3	Logistic regression	93%	0.91	0.94	0.95	0.92	0.97	0.95
	Linear SVC	93%	0.90	0.94	**0.96**	0.90	0.97	0.94
	GNB	93%	0.90	0.94	0.95	0.91	0.97	0.92
	Random Forest	92%	0.89	0.94	0.90	0.93	0.98	0.95
	AdaBoost	**94%**	**0.91**	**0.95**	0.93	**0.94**	**0.98**	**0.96**
	RUSBoost	92%	0.90	0.94	0.92	0.92	0.97	0.9
	KNN	93%	0.90	0.94	0.92	0.93	0.96	0.94
	Mixture of experts	92%	0.89	0.94	0.87	0.94	0.98	0.95
	Snapshot ensembling	93%	0.91	0.94	0.97	0.91	0.97	0.95
M4	Logistic regression	64%	0.00	0.78	0.00	1.00	0.54	0.34
	Linear SVC	50%	0.33	0.61	0.33	0.60	0.58	0.36
	GNB	69%	0.69	0.70	**0.93**	0.56	0.90	0.87
	Random Forest	90%	0.86	0.92	0.84	**0.93**	**0.97**	**0.95**
	AdaBoost	**91%**	**0.88**	**0.93**	0.90	0.92	0.94	0.90
	RUSBoost	88%	0.85	0.90	0.88	0.87	0.96	0.94
	KNN	74%	0.66	0.79	0.69	0.78	0.85	0.75
	Mixture of experts	89%	0.85	0.91	0.86	0.90	0.97	0.94
	Snapshot ensembling	64%	0.0	0.78	0.0	1.00	0.55	0.35
M5	Logistic regression	**95%**	0.93	**0.96**	0.97	0.94	**0.98**	0.91
	Linear SVC	**95%**	**0.94**	**0.96**	**0.99**	0.93	0.95	0.84
	GNB	95%	0.93	0.96	0.94	0.95	0.96	0.87
	Random Forest	**95%**	0.93	**0.96**	0.97	0.94	**0.98**	0.95
	AdaBoost	95%	0.93	**0.96**	0.97	0.93	0.97	0.90
	RUSBoost	94%	0.92	0.95	0.96	0.93	0.97	0.89
	KNN	95%	0.93	0.96	0.95	0.94	0.98	**0.96**
	Mixture of experts	94%	0.92	0.95	0.94	0.94	0.98	0.90
	Snapshot ensembling	93%	0.91	0.94	0.99	0.90	0.98	0.93
M6	Logistic regression	93%	0.91	0.94	0.97	0.90	0.98	0.97
	Linear SVC	89%	0.86	0.91	0.88	0.90	0.97	0.95
	GNB	89%	0.86	0.90	**1.00**	0.82	0.92	0.72
	Random forest	95%	**0.94**	0.96	0.92	0.97	**0.99**	**0.99**
	AdaBoost	**96%**	**0.94**	**0.97**	0.92	**0.98**	**0.99**	**0.99**
	RUSBoost	93%	0.91	0.95	0.92	0.94	0.99	0.98
	KNN	92%	0.89	0.94	0.89	0.94	0.97	0.96
	Mixture of experts	94%	0.91	0.95	0.89	0.97	0.99	0.99
	Snapshot ensembling	88%	0.83	0.91	0.80	0.92	0.95	0.90
M7	Logistic regression	96%	0.94	0.97	0.93	0.97	0.99	0.99
	Linear SVC	97%	0.96	0.97	0.95	**0.98**	**1.00**	**1.00**
	GNB	86%	0.83	0.88	0.95	0.81	0.98	0.97
	Random Forest	96%	0.95	0.97	0.93	0.98	1.00	0.99
	AdaBoost	**98%**	**0.97**	**0.98**	**0.97**	**0.98**	**1.00**	**1.00**
	RUSBoost	97%	0.96	0.98	0.95	0.98	1.00	**1.00**
	KNN	96%	0.94	0.97	0.95	0.96	0.99	0.98
	Mixture of experts	**98**%	**0.98**	**0.99**	**0.98**	**0.98**	**1.00**	**1.00**
	Snapshot ensembling	85%	0.79	0.89	0.75	0.92	0.94	0.88

The comparative analysis of these models highlights the effectiveness of various preprocessing and feature extraction techniques in enhancing classification accuracy. The performance of different ML classifiers varied based on the preprocessing techniques applied. LR demonstrated high accuracy (96%–98%) with simpler preprocessing methods such as green channel extraction and vessel removal, maintaining strong F1‐scores for both normal and fungal classes. However, its accuracy dropped to approximately 64% when more complex preprocessing techniques like histogram modelling and image normalization were used. The linear SVC classifier showed consistent performance across various preprocessing types, achieving up to 97% accuracy with high F1‐scores when illumination and adaptive histogram equalization were applied. However, its accuracy declined (50%–64%) with more complex preprocessing. GNB exhibited moderate accuracy, ranging from 46% with extensive normalization to around 95% when using simpler preprocessing techniques like green channel extraction and CLAHE. F1‐scores remained high (around 0.90) for both classes in high‐accuracy configurations. Random Forest achieved strong performance, peaking at 99% accuracy with simpler preprocessing, while maintaining balanced F1‐scores across both classes. However, accuracy dropped to 63%–90% with more advanced preprocessing, showing variability based on feature extraction methods. AdaBoost performed well across different setups, with accuracy ranging from 94‐98% and F1‐scores near 0.98 in high‐performing configurations. It performed best when illumination equalization and vessel removal were used, though accuracy slightly decreased with complex preprocessing. RUSBoost exhibited performance comparable to AdaBoost, reaching an accuracy of approximately 97% in simpler setups while maintaining balanced F1‐scores across classes. However, results were slightly lower (66‐88%) when complex preprocessing and feature extraction methods were applied. kNN demonstrated moderate to high accuracy (92%–95%) with simpler preprocessing techniques, though accuracy declined to 68%–74% when using advanced feature extraction methods.

The MoE ensemble model achieved high accuracy (98%) in simpler preprocessing settings (M1), with balanced F1‐scores (0.98–0.99) and perfect AUROC/AUPRC (1.0, 1.0), indicating excellent classification performance. However, in complex preprocessing setups like M2, its accuracy dropped to 64%, showing reduced sensitivity (0.54) for fungal detection. While MoE excels in structured environments, its performance declines with noisy or complex feature extraction, similar to other classifiers. Snapshot ensembling achieved its best performance in M3, with 94% accuracy, AUROC of 0.97, and AUPRC of 0.94, closely matching AdaBoost. Across all configurations, it consistently delivered strong results but was generally outperformed by RF, AdaBoost, and MoE, which reached up to 99% accuracy. While effective in preventing overfitting, its impact was dataset‐dependent and less dominant than other ensemble methods.

**FIGURE 4:**
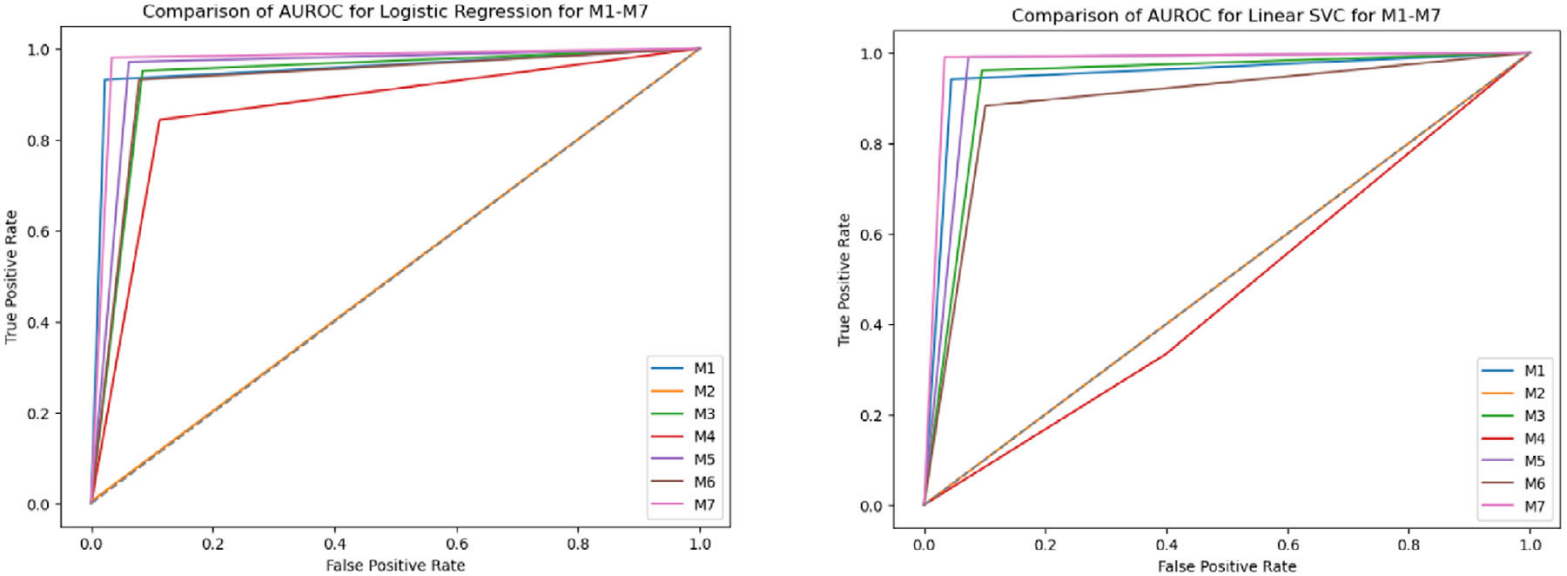
AUROC performance of M1 to M7 approaches considered for the experiments: (a) linear regression, (b) linear SVC.

**FIGURE 5:**
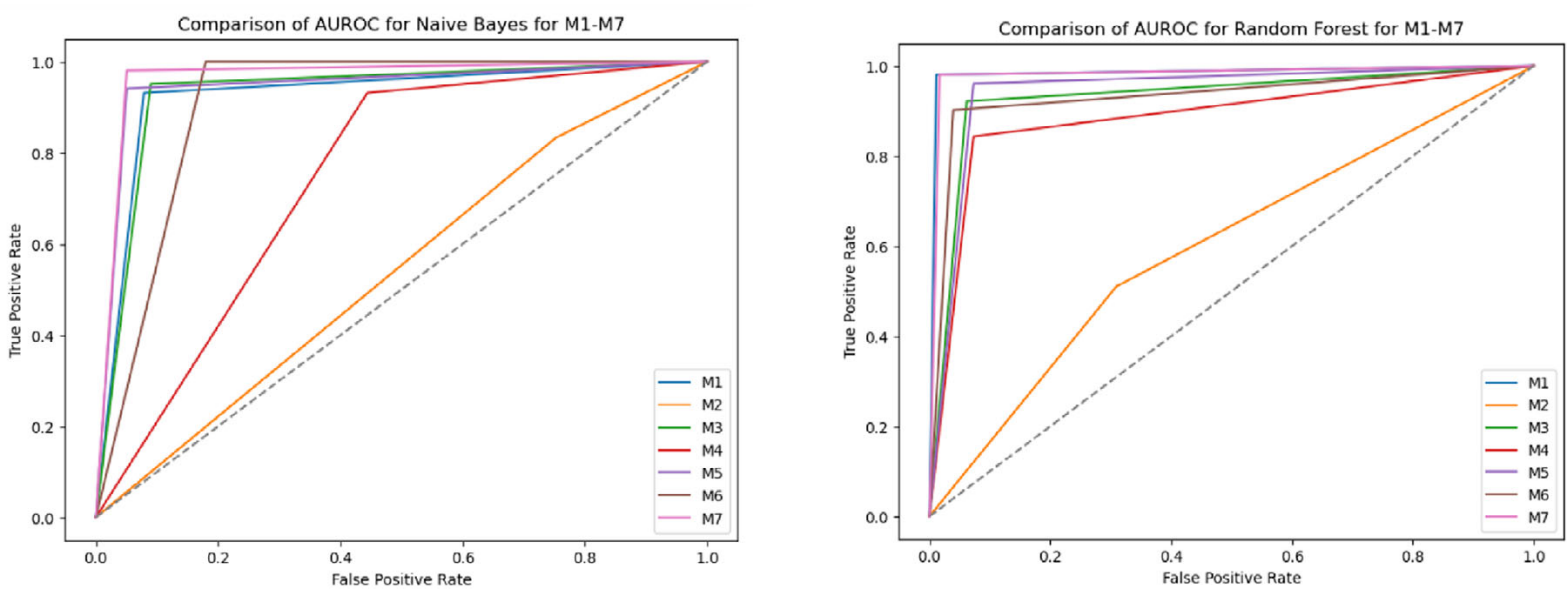
AUROC performance of M1 to M7 approaches considered for the experiments: (a) Gaussian Naive Bayes, (b) Random Forest.

**FIGURE 6:**
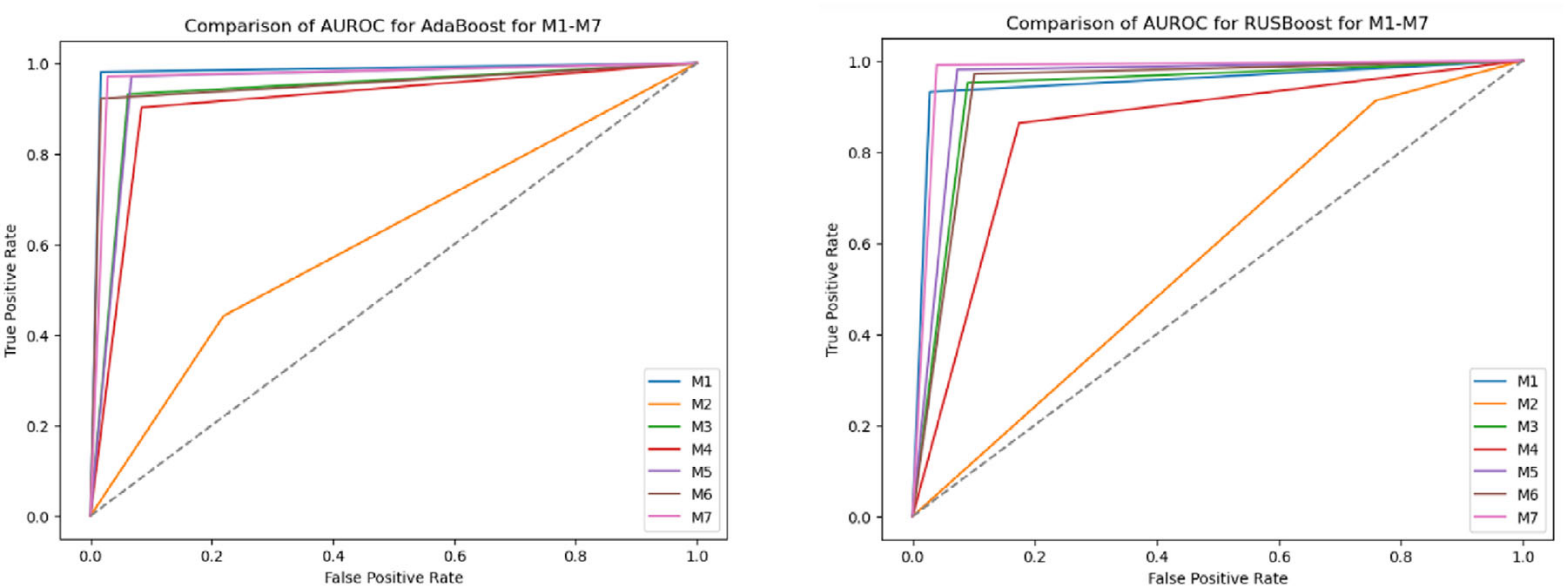
AUROC performance of M1 to M7 approaches considered for the experiments: (a) AdaBoost, (b) RUSBoost.

**FIGURE 7:**
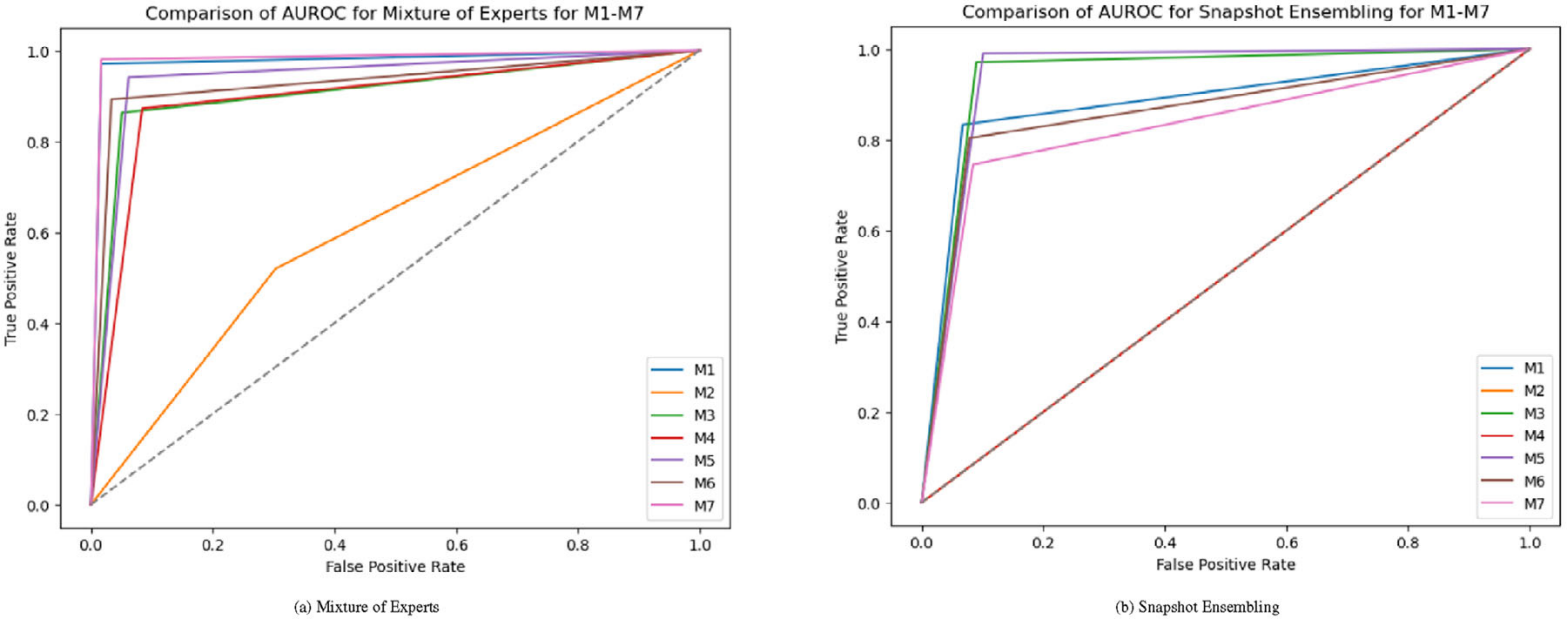
AUROC performance of M1 to M7 approaches for the ensemble strategies considered: (a) mixture of experts, (b) snapshot ensembling.

The performance analysis of various ML models highlights the significant impact of preprocessing and feature extraction on classification accuracy in detecting FK. The AUROC performance of all seven model groups (M1‐M7) for all the classifiers is shown in Figures 4, 5, 6, 7. Notably, applying green channel preprocessing alongside a feature set of twelve characteristics resulted in the highest accuracy of 99% with the RF model, demonstrating the effectiveness of these features in capturing essential image details. The MoE model also performed exceptionally well, achieving 98% accuracy with balanced F1‐scores (0.98–0.99) and perfect AUROC/AUPRC scores (1.0, 1.0), underscoring its effectiveness in structured preprocessing environments. Snapshot ensembling exhibited strong performance, with its best result reaching 94% accuracy (M3) and an AUROC of 0.97, showing its capability to generalize well while mitigating overfitting. However, it was generally outperformed by RF, AdaBoost, and MoE, which consistently achieved superior classification results. In contrast, the use of more complex techniques such as image normalization and histogram modelling led to a decline in accuracy to 64% for both RF and MoE, suggesting that these methods may not sufficiently emphasize the critical features needed to distinguish between normal and fungal conditions. Additionally, models such as AdaBoost and RUSBoost exhibited consistent robustness across various feature extraction methods, achieving high F1‐scores, particularly for fungal detection. This highlights their adaptability to the imbalanced datasets frequently encountered in medical imaging. Techniques incorporating convolution with a Gaussian mask and local maximum region extraction also produced promising results, maintaining high accuracy of approximately 93% for models like LR and Linear SVC.

### 4.3 Visualization of ML Model Performance

**FIGURE 8:**
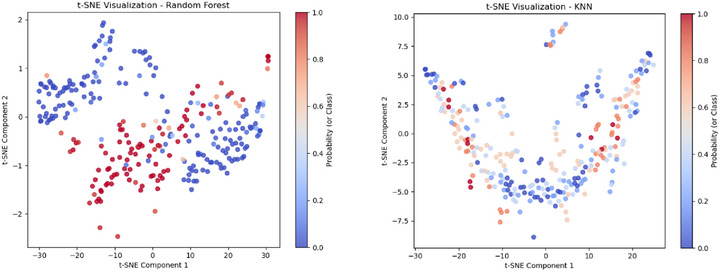
t‐SNE plots for: (a) Random Forest for M1, (b) KNN for M2.

The t‐SNE plot for the RF model in M1, shown in Figure 8a, demonstrates effective class separability, with distinct clustering of data points corresponding to different probability values. The presence of a transition region, where blue and red points intermingle, suggests decision boundary complexities, likely due to feature overlap in certain instances. The well‐spread distribution across both t‐SNE components indicates that the extracted features contribute to a robust representation, aligning with the high classification accuracy achieved. However, the intermediate gradient regions highlight areas where the model exhibits lower confidence, potentially impacting misclassification rates. The t‐SNE plot for KNN in M2, shown in Figure 8b, exhibits a more dispersed clustering pattern compared to RF in M1, indicating that KNN struggles with clear class separability. The overlapping blue and red regions suggest significant decision boundary ambiguity, likely due to KNN's reliance on local neighbourhood structures, which can be sensitive to variations in feature distributions. The presence of interspersed probability values (gradient areas) highlights KNN's lower confidence in classification, particularly in complex regions with high feature overlap.

**FIGURE 9:**
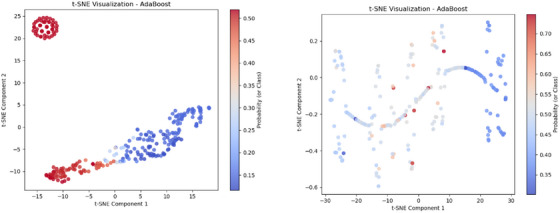
t‐SNE plots for: (a) AdaBoost for M3, (b) AdaBoost for M4.

The t‐SNE visualization for AdaBoost in M3, shown in Figure 9a, presents well‐separated clusters, indicating strong class separability. The compact red cluster at the top‐left suggests a group of instances classified with high confidence, while the gradient transition in the central region reflects areas of moderate classification confidence. The more dispersed blue region implies slight uncertainty in those instances. This pattern aligns with AdaBoost's robust performance, as it consistently maintains high accuracy even when complex preprocessing techniques are applied. The t‐SNE visualization for AdaBoost in M4, shown in Figure 9b, reveals a structured yet complex decision boundary, indicating that the classifier effectively captures intricate feature interactions. The curved and dispersed clusters suggest that M4's advanced preprocessing techniques, including adaptive contrast adjustment and optic disk removal, contribute to enhanced feature separability. The probability gradient from red to blue highlights areas of high classification confidence, though some regions display mixed probabilities, indicating uncertainty. Compared to simpler models, M4's AdaBoost classifier demonstrates improved adaptability while still exhibiting areas where class overlap leads to lower confidence.

**FIGURE 10:**
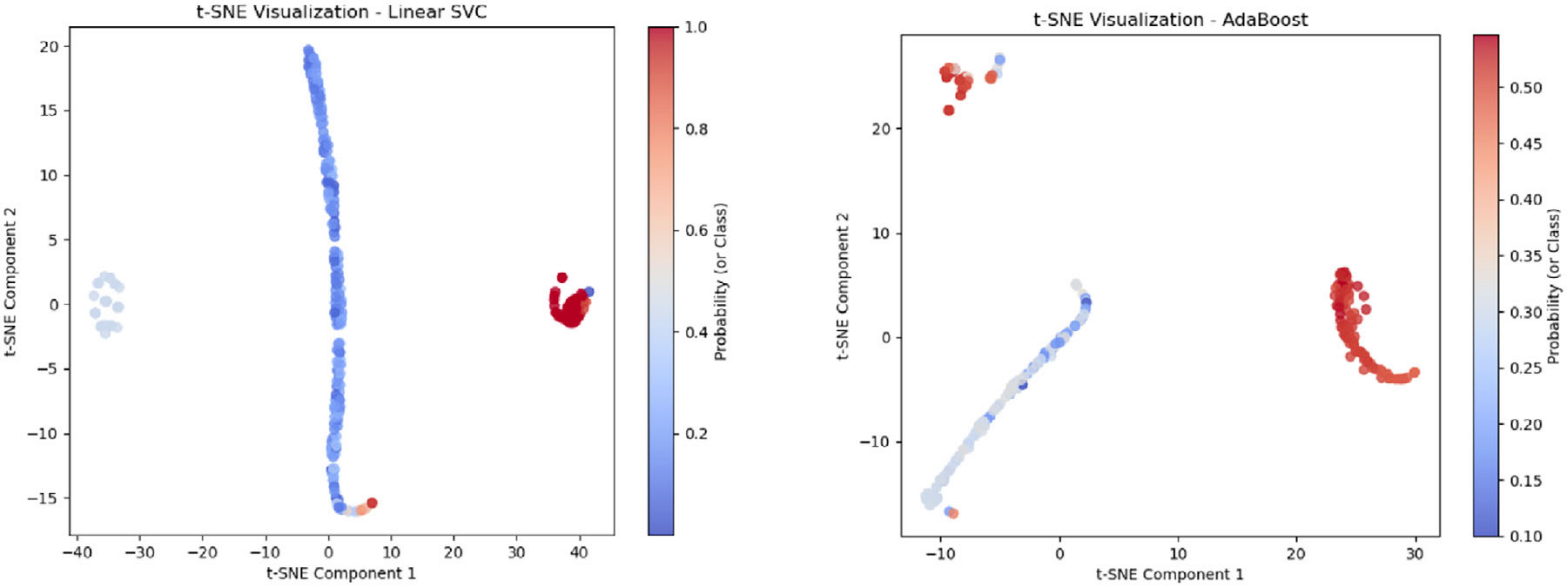
t‐SNE plots for: (a) Linear SVC for M5, (b) AdaBoost for M6.

**FIGURE 11:**
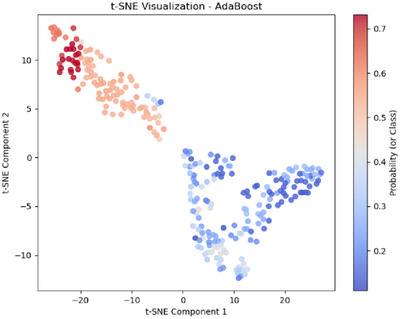
t‐SNE plots for AdaBoost for M7.

The t‐SNE visualization for Linear SVC in M5, shown in Figure 10a, displays three well‐separated clusters, indicating effective class differentiation. The vertical distribution of the major blue cluster suggests that certain feature dimensions play a strong role in class separation. The red cluster on the right represents high‐confidence classifications, while the scattered blue cluster on the left indicates potential misclassified or uncertain samples. This visualization highlights the effectiveness of M5's feature extraction techniques in enhancing class separability, though some degree of overlap remains. The t‐SNE visualization for AdaBoost in M6, shown in Figure 10b, presents two well‐separated clusters, indicating effective class differentiation. The rightmost red cluster signifies high‐confidence classifications, while the more dispersed leftmost blue cluster suggests some uncertainty or misclassified samples. The gradual transition in probability reflects varying classifier confidence, demonstrating AdaBoost's adaptability to the extracted features while highlighting areas where classification certainty varies. Similar observations can be made from Figure 11 for AdaBoost in M7, where only a few blue points appear at the edge of the leftmost red cluster, indicating possible boundary overlap.

**FIGURE 12:**
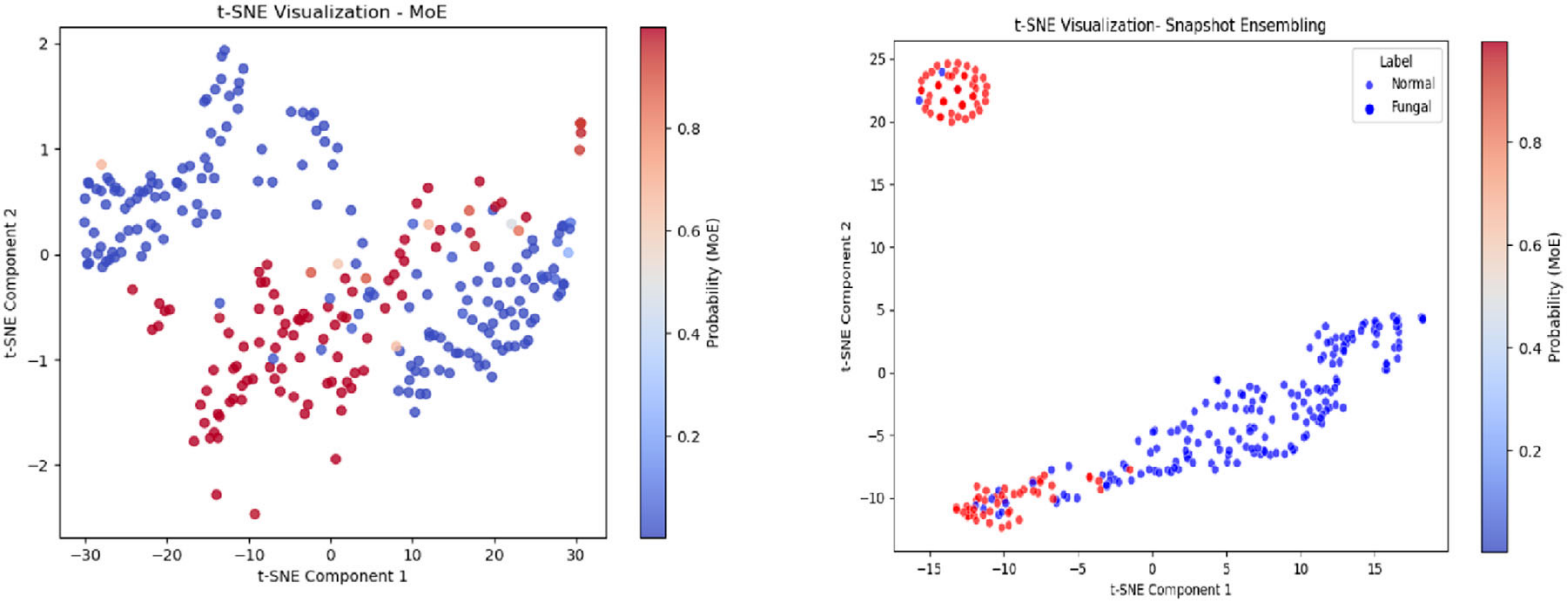
t‐SNE plots for: (a) mixture of experts for M1, (b) snapshot ensembling for M3.

The t‐SNE plot for the mixture of experts (MoE) in M1 in Figure 12a illustrates a strong degree of class separability, with data points forming distinct clusters based on their probability values. The colour gradient represents the probability output of the MoE model, transitioning smoothly between blue (low probability) and red (high probability). The presence of an intermixed region, where blue and red points overlap, highlights decision boundary complexities, possibly due to feature similarities in certain cases. The broad dispersion along both t‐SNE components suggests that the extracted features contribute to a well‐structured representation, supporting the model's predictive capability. However, the presence of soft transition zones, where probabilities are neither extreme, indicates areas where the model exhibits uncertainty, which may contribute to misclassification.

The t‐SNE plot for the snapshot ensembling in M3 in Figure 12b demonstrates clear class separability between normal and fungal samples. The blue points (normal) and red points (fungal) mostly form distinct clusters, indicating that the extracted features effectively capture the differences between the two categories. Notably, there is a well‐isolated cluster of fungal cases in the upper‐left corner, suggesting a strong feature distinction in these samples. However, in the lower‐left region, there is some intermixing of normal and fungal samples, indicating a potential decision boundary complexity where the model may exhibit uncertainty. The elongated spread of normal cases along the t‐SNE components suggests a diverse representation within this class, whereas the fungal cases remain relatively compact. This pattern aligns with the expectation that fungal infections have more distinct feature characteristics, whereas normal cases may exhibit more variation, potentially leading to classification challenges in borderline cases.

### 4.4 Ablation Study

We performed an ablation study to assess the impact of individual preprocessing techniques and feature extraction methods on classification performance. By systematically removing or modifying specific components, we aim to evaluate their contributions to model accuracy and robustness, to identify essential preprocessing steps that enhance feature representation while also detecting techniques that may introduce noise or redundancy. The detailed findings from the ablation experiments conducted across different preprocessing models (M1–M7) are presented in this section, highlighting key observations regarding feature significance and classification performance.

The ablation study underscored the importance of image normalization, as its removal led to accuracy values dropping to baseline levels, highlighting its role in standardizing features. For instance, M1, which used only the green channel as a preprocessing technique, achieved 99% accuracy with the RF classifier, identifying area, perimeter, and compactness as the most significant features. In contrast, M2 exhibited accuracies ranging from 46% to 68%, with ensemble methods performing slightly better. The most influential features in M2 included brightness, greyscale standard deviation, and mean pixel values. Conversely, eliminating histogram modelling improved accuracy to 94%–96%, suggesting that it may introduce noise or contribute to overfitting. M3 initially demonstrated strong performance across classifiers, with accuracies between 92% and 94%, where maximum and mean peak and ramp heights were identified as key features. However, the ablation study revealed that removing local maximum region extraction significantly reduced accuracy to 64%–67%, emphasizing its critical role in feature enhancement. In contrast, eliminating Gaussian mask convolution resulted in only a slight decline in accuracy to 91%–93%, indicating that while it contributes to feature refinement, its impact is relatively less significant.

The performance of M4 ranged from 50% to 91%, with significant variations across classifiers, where area and eccentricity were identified as the most important features. The ablation study revealed that removing preprocessing techniques such as spatial calibration, illumination equalization, and denoising had a moderate impact on accuracy, particularly for logistic regression and SVC, while RF, AdaBoost and mixture of experts remained robust. Eliminating techniques like optic disk removal, the green channel, and dynamic enhancement also affected accuracy, especially for SVC. In contrast, M5 achieved accuracy of 94%–95% across classifiers, with standard intensity, mean intensity, mean area and perimeter as the most influential features. Interestingly, removing vessel removal improved accuracy, with LR and SVC reaching 99%, suggesting possible overprocessing. Median filtering and mathematical morphology had minimal impact, whereas excluding adaptive contrast slightly reduced performance, indicating its moderate importance. M6 achieved accuracies of 89%–96% across classifiers, with area, perimeter, and aspect ratio as the key features. The removal of background noise reduction led to significant accuracy drops for LR (86%) and GNB (66%), highlighting its critical role. Excluding MA extraction affected SVC and KNN while boosting RF and ensemble methods. Non‐maximum suppression and region growing had minimal effects, maintaining stable performance. M7 showed accuracies of 86%–98% across classifiers, where centroid distance, area, standard deviation, and the mean of Canny edges were the most important features. Interestingly, removing illumination equalization improved most classifiers to 99%, indicating potential redundancy. Excluding adaptive histogram equalization or grey normalization caused minimal changes, suggesting limited individual impact. However, removing MA candidate extraction improved GNB (96%) while maintaining stable performance across other models.

## 5 Discussion

The application of the proposed models in clinical ophthalmology could significantly improve the diagnosis and management of fungal keratitis, as any delay or inaccurate detection often leads to severe visual impairment. Integration into routine workflows may involve embedding the models into slit‐lamp or confocal microscopy imaging systems to provide immediate, image‐based decision support at the point of care. Such tools could assist ophthalmologists by highlighting suspicious lesions, suggesting likelihood scores for FK, and offering a second opinion in diagnostically challenging cases. This would be particularly valuable in resource‐limited or high‐volume clinical settings, where access to corneal specialists may be restricted. From a workflow perspective, automated FK screening could streamline triage by rapidly distinguishing fungal from non‐fungal keratitis, ensuring timely initiation of antifungal therapy. Beyond diagnostic support, integration with electronic health records could facilitate longitudinal monitoring and enable more consistent documentation. Nevertheless, successful deployment in practice would depend on validation across diverse patient populations, interpretability of model outputs, and acceptance by ophthalmologists who must trust that the system enhances rather than disrupts their clinical decision‐making.

In discussing the implications of this work, it is also important to acknowledge certain limitations and considerations that may affect the generalizability, clinical deployment, and practical integration of the proposed framework. These factors are outlined below.

- Our study utilizes a single dataset, which can potentially limit its generalizability. Also, we assume that the IVCM dataset accurately represents the target patient population and that the data labels are reliable, for the purposes of this study. The performance of different ML models is also dependent on the preprocessing methods applied (M1 to M7). To strengthen the robustness and real‐world applicability of the proposed models, future research should incorporate multi‐centre and multi‐source datasets.
- Although the proposed methodology performs well on the IVCM dataset, the real‐world clinical deployment requires external validation by ophthalmologists and domain experts from the corneal medicine specialty. Expert review is essential to assess model performance in diverse clinical scenarios and ensure that the predictions align with clinical reasoning and standards. Our future work will involve validation studies in collaboration with clinical experts to assess usability and interpretability, and facilitate the integration into real‐world decision‐making workflows.
- For our study, an open‐source dataset was used, hence, the regulatory compliance related to patient's data privacy is not directly applicable. But, this will be major concern in real‐world clinical settings, requiring architectures like federated learning to ensure a privacy‐aware, multi‐centre learning framework. Additionally, practical aspects such as scalability and interoperability with existing clinical workflows need to be addressed for effective real‐world use of the designed models in clinical settings.

## 6 Conclusion and Future Work

In this study, we comprehensively evaluated various ML models and ensemble techniques for the task of FK classification. Extensive experimentation with various preprocessing methods, feature extraction techniques, and ML models demonstrated that an optimized combination of preprocessing strategies and advanced ensemble model architectures enhances diagnostic reliability and robustness. Techniques such as green channel isolation, adaptive histogram equalization, Gaussian blur, and illumination equalization significantly improved image quality and feature clarity, directly impacting classification performance. Among conventional ML techniques, SVM and ensemble models like RUSBoost emerged as top performers, particularly when combined with advanced feature extraction methods. While ML‐based approaches have shown promising results, they also present certain limitations. Traditional ML models rely heavily on handcrafted features, which may not fully capture the complex patterns present in FK images. Additionally, most ML models often struggle with domain shifts when applied to diverse datasets acquired under different imaging conditions. Another concern is that explainability needs to be incorporated, particularly with ensemble methods, which often function as black‐box models, limiting their interpretability for clinical applications.

A key takeaway from this study is the critical role of targeted preprocessing and feature engineering in addressing clinical variability within datasets, thereby improving model generalization. The findings highlight that robust preprocessing techniques, such as CLAHE, adaptive thresholding, and non‐maximum suppression, effectively mitigate challenges posed by diverse imaging conditions and noise in clinical data. Furthermore, incorporating advanced evaluation metrics, such as the F1‐score alongside accuracy, provides a more comprehensive assessment of model performance. This is particularly important in medical diagnostics, where precision is essential. Future research will address these limitations by exploring DL models, which can automatically learn hierarchical feature representations, potentially reducing the reliance on handcrafted features. Efforts will also focus on refining preprocessing techniques to better accommodate heterogeneous datasets, thereby enhancing model generalization across diverse imaging conditions. Our approach demonstrates promising performance, however, the experiments were conducted only a single dataset. Relying on one dataset may limit the generalizability of the findings to a more general, diverse population. Future work will validate the model using external datasets from different institutions to make it more general and suitable for clinical applications. Additionally, integrating advanced AI models that incorporate multimodal data, such as clinical history and additional imaging modalities, is expected to improve diagnostic accuracy by providing a more comprehensive perspective on each case. Enhancing model explainability will be a key priority to foster clinician trust and ensure transparency in diagnostic recommendations. Furthermore, rigorous validation across diverse clinical environments will be essential to ensuring the practical applicability, robustness, and scalability of these diagnostic models in real‐world settings.

## Author Contributions

**Sowmya Kamath S**.: conceptualization, data curation, formal analysis, investigation, methodology, project administration, resources, supervision, validation, visualization, writing – original draft, writing – review and editing. **Shikha Reji**: conceptualization, investigation, methodology, software, validation, visualization, writing – original draft, writing – review and editing. **Vaibhava Lakshmi**: conceptualization, investigation, methodology, software, validation, visualization, writing – original draft, writing – review and editing. **Supreetha R**.: conceptualization, investigation, methodology, software, validation, visualization, writing – original draft, writing – review and editing. **Pratiksha Gawas**: conceptualization, investigation, methodology, software, visualization, writing – original draft, writing – review and editing. **Veena Mayya**: conceptualization, investigation, writing – review and editing. **Manali Hazarika**: validation, writing – review and editing.

## Funding

The authors have nothing to report.

## Conflicts of Interest

The authors declare no conflicts of interest.

## Data Availability

The data that support the findings of this study are openly available in the IVCM Keratitis dataset from Figshare repository, https://figshare.com/articles/dataset/Dataset/19838083.
